# Transcriptional analysis of *Kluyveromyces marxianus* for ethanol production from inulin using consolidated bioprocessing technology

**DOI:** 10.1186/s13068-015-0295-y

**Published:** 2015-08-14

**Authors:** Jiaoqi Gao, Wenjie Yuan, Yimin Li, Ruijuan Xiang, Shengbo Hou, Shijun Zhong, Fengwu Bai

**Affiliations:** School of Life Science and Biotechnology, Dalian University of Technology, Dalian, 116024 China; School of Life Science and Biotechnology, Shanghai Jiaotong University, Shanghai, 200240 China

**Keywords:** *Kluyveromyces marxianus*, RNA-seq, Ethanol fermentation, Inulin, Transcriptome analysis

## Abstract

**Background:**

Ethanol production from non-crop materials, such as Jerusalem artichokes, would make a great contribution to the energy industry. The non-conventional yeast, *Kluyveromyces marxianus*, is able to carry out ethanol fermentation of sugar molecules obtained from inulin-containing materials by consolidated bioprocessing. Lower inulin concentrations and micro-aeration can lead to a relatively fast and ideal fermentation process; however, it is unclear what causes the inhibition of higher concentrations of inulin and the promotion effect of aeration.

**Results:**

Next-generation sequencing technology was used to study the global transcriptional response of *K. marxianus* Y179 under three fermentation conditions, including 120 g/L inulin without aeration (120-N), 230 g/L inulin without aeration (230-N), 230 g/L inulin with aeration by ORP controlling at −130 mV (230-130mV). A total of 35.55 million clean reads were generated from three samples, of which 4,820 predicted that open reading frames were annotated. For differential expression analysis, 950 and 1,452 differentially expressed genes were discovered under the conditions of 230-130mV and 120-N, respectively, and the sample 230-N was used as the control. These genes are mainly associated with the pathways of central carbon metabolism and ethanol formation. Increased expression of inulinase and the low activity of the autophagy-related gene, *ATG8*, ensured fast and ideal fermentation processes.

**Conclusions:**

Despite being reported as the “crabtree-negative” species, *K. marxianus* Y179 could achieve favorable ethanol fermentation profiles under micro-aeration and high inulin concentrations. *K. marxianus* Y179 cells responded to inulin concentrations and micro-aeration that is involved in the whole ethanol metabolism network. These results will serve as an important foundation for further exploration of the regulatory mechanisms involved in ethanol fermentation from inulin by consolidated bioprocessing and also provide a valuable reference for future studies on optimization and reconstruction of the metabolism network in *K. marxianus.*

**Electronic supplementary material:**

The online version of this article (doi:10.1186/s13068-015-0295-y) contains supplementary material, which is available to authorized users.

## Background

The production of bioethanol from non-grain materials will be an important developmental trend in the fuel ethanol industry, especially in a country like China with a large population and relatively few arable lands. Recently, some inulin-containing materials, especially Jerusalem artichokes, have received increasing attention for their advantages, such as high yield, resistance to poor soil, drought, low temperatures, and pests [1–3]. Inulin in the roots and tubers of these plants, which is linked by fructose via a β-2,1 bond [4], is usually regarded as the main carbohydrate for ethanol fermentation.

*Kluyveromyces marxianus*, a sister species of *Kluyveromyces lactis*, is another type of “non-conventional” yeast that has been widely studied and has also been reported as a model of “crabtree-negative” yeast, in contrast to *Saccharomyces cerevisiae* [5]. Due to its high temperature resistance, rapid growth, and capacity to utilize various substrates, there are increasing applications of *K. marxianus* in biofuel industrial biotechnology. Furthermore, *K. marxianus* has been proven to be a key strain that can achieve ethanol fermentation from Jerusalem artichokes by consolidated bioprocessing technology (CBP), which combines inulinase production, inulin hydrolysis, and ethanol production [2, 6, 7].

The previous results of ethanol fermentation from the inulin of Jerusalem artichokes showed some problems under high inulin concentration conditions, such as residual inulin and a long fermentative time [7]. However, these problems did not happen under micro-aeration or lower inulin concentration conditions (our unpublished data). The molecular mechanisms of the inhibition of higher inulin concentrations and the effects of aeration are still unclear.

Although the whole-genome sequences of *K. marxianus* were first published in 2012 [8], publications on the transcriptional analysis of *K. marxianus* are very limited. Lertwattanasakul et al. [9] conducted complete genome sequencing and transcriptome analyses of *K. marxianus* DMKU 3-1042 to identify genes related to growth at a high temperature and the utilization of various substrates, especially xylose and arabinose. However, there have been no other reports on related information, especially ethanol fermentation by *K. marxianus*.

Considering the preponderance of a low background noise, less amount of RNA required, and the low cost involved in mapping the transcriptome of large genomes [10], in this article, the next-generation sequencing technology for RNA (RNA-seq) was utilized to study the global transcriptional response of *K. marxianus* Y179. The transcriptional response of *K. marxianus* Y179 was studied under various ethanol fermentative conditions [that includes low (12%) to high (23%) inulin concentration and in conditions ranging from anaerobic to micro-aeration].

## Results and discussion

### Physiological diversity for ethanol fermentation among various *K. marxianus* strains

Though *S. cerevisiae* has been widely applied in industrial ethanol production, *K. marxianus* is now attracting an increasing amount of attention for its specific physiological diversity. As shown in Table 1, *K. marxianus* can use multiple substrates, even xylose and lactose, to produce ethanol. Moreover, although *K. marxianus* was known to have a poor ethanol tolerance compared to *S. cerevisiae* [12], the maximum ethanol concentration for *K. marxianus* fermentation broth is more than 100 g/L. Despite the differences of substrates [6, 11–18], the higher specific growth rates of *K. marxianus* guarantee superior results for various fermentative processes.

**Table 1:** Physiological analysis of ethanol production within different *K. marxianus* strains

Strains	Substrates	Concentrations (g/L)	Duration (h)	μ_m_ (h^−1^)	E_m_ (g/L)^a^	I_m_ (U/mL)^b^	References
KM KD-15	Sucrose + lactose	200	72	–	104	–	[11]
KM UFV-3	Lactose	170	–	–	80	–	[12]
KM M15	Molasses	150	48	0.3	70	–	[13]
KM DMKU 3-1042	Cane juice	220	60	–	87	–	[14]
KM CE025	Glucose + xylose	28 + 30	72	0.0519	12	–	[15]
KM CBS 7858	Lactose	20	10	0.87	6	–	[16]
KM ATCC 8554	Inulin	153	60	–	61	3	[6]
KM Y179	Inulin	232	72	0.27	93	17.5	This study
	Inulin	227	36	0.342	98	22.9	This study
	Inulin	114	24	0.474	51	25.6	This study
KM CBS 6556	Sucrose	10	9	0.7	–	1.5	[17]
	Glucose	10	10	0.495	–	–	[18]

^a^Maximum ethanol concentration.

^b^Maximum inulinase activity.

### Ethanol fermentation by *K. marxianus* Y179 from inulin

Batch fermentation was performed to investigate the differences of gene expression between high and low inulin concentrations and from anaerobic conditions to micro-aeration. Higher concentrations of inulin repressed the whole process, but aeration promoted it [19, 20]. Therefore, 120 and 230 g/L were selected as low and high inulin concentrations, respectively. An appropriate aeration, by controlling ORP at −130 mV [19], was set as the aeration condition for transcriptional analysis. The samples were taken at their own end of fermentation to erase the complicated chemical and biological changes that occurred during the fermentation process.

**Fig. 1:**
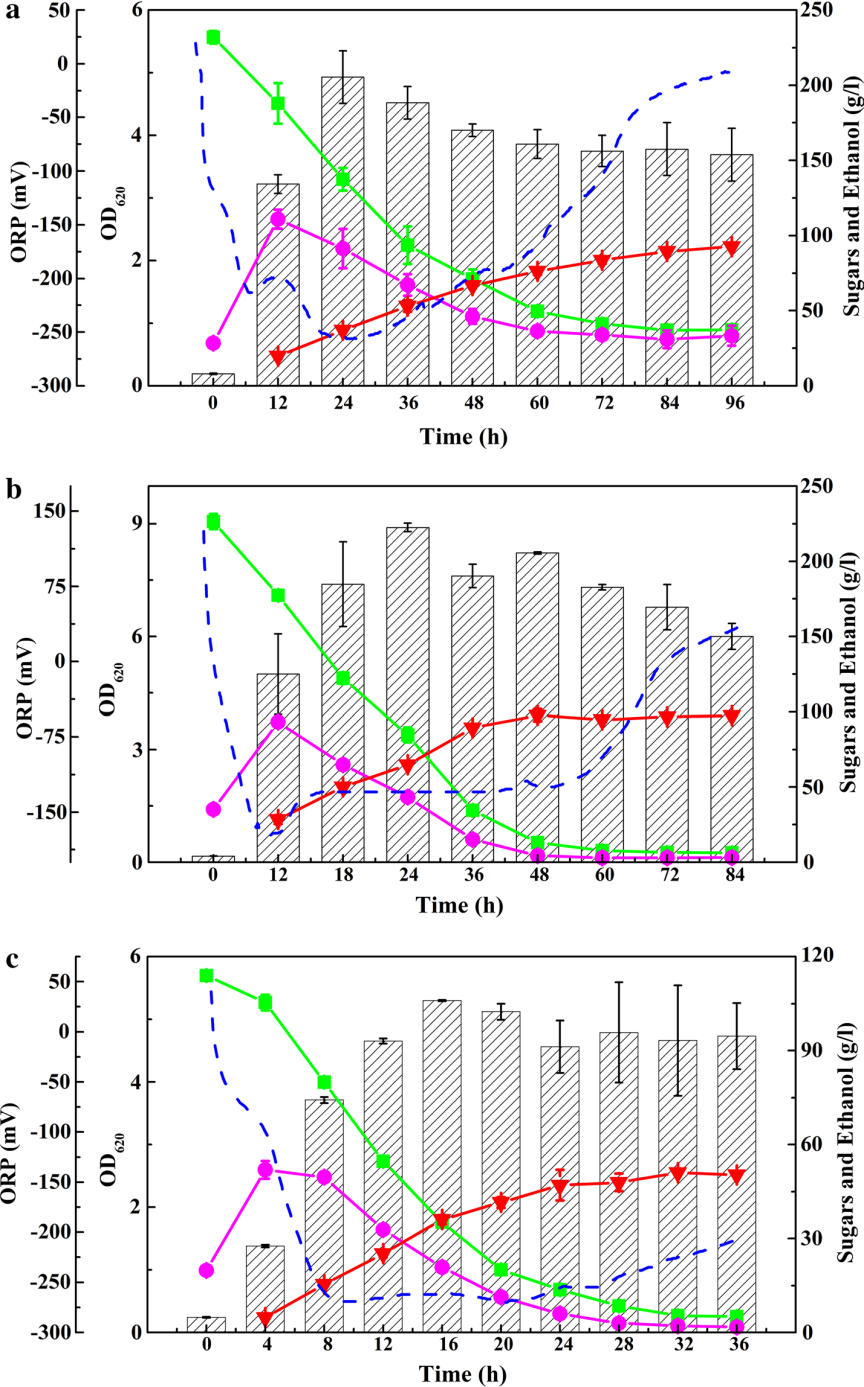
Profiles of fermentation under three different conditions by *K. marxianus.* Seed culture (100 mL) was inoculated into a 3-L fermenter with a 1.0-L working volume. **a** 230-N-72. **b** 230-130mV-36. **c** 120-N-24. OD_620_ (*square with crossed lines*), total sugar (*green colored square*), reducing inulin (*purple colored circle*) and ethanol (*red colored inverted triangle*), ORP (*blue colored dashed lines*).

The physiology of ethanol fermentation by *K. marxianus* Y179 under three conditions are shown in Table 1 and Fig. 1. As reported previously [19, 20], *K. marxianus* Y179 showed obvious catabolite repression under high inulin concentrations. Micro-aeration (ORP −130 mV) allowed *K. marxianus* Y179 to achieve the maximum ethanol concentration from inulin reported so far. The relatively high expression of inulinase by *K. marxianus* Y179 was in accordance with a previous study [20]. Consequently, the transcriptional analysis was conducted to investigate the differences of gene expression between high inulin and low inulin concentrations, and from anaerobic conditions to micro-aeration conditions.

### Genome annotation and overall gene expression analysis by RNA-seq

#### Genome annotation

Genome sequencing of *K. marxianus* had already been completed in 2012 [8]. The 10.9 Mb genome of *K. marxianus* var. m*arxianu*s KCTC 17555 (=CBS 6556 = ATCC 26548) was obtained via the Illumina sequencing platform, which contained 40.1% guanine and cytosine. Hence, the genome sequence of *K. marxianus* KCTC 17555 was employed as the reference genome for RNA-seq in this study. However, no annotation information for *K. marxianus* KCTC 17555 is currently available on the web site, so the genome sequence obtained by Jeong et al. was annotated according to the information from the Gene Ontology (GO) database and the Kyoto Encyclopedia of Genes and Genomes (KEGG) database. The existing genome sequence was integrated into eight chromosomes and the mitochondrial genome. We obtained 2,472 annotations from the GO database, while 1,880 functional genes were obtained from the KEGG database. This annotation information provided a good foundation as a reference for RNA-seq (Table 2).

**Table 2:** Overview of genomes of *K. marxianus* KCTC 17555 and annotations by GO and KEGG databases

	Length (Mb)	CDS (GO)	CDS (KEGG)		CDS length (bp)			
			With annotation	Without annotation	500	501–1,000	1,001–2,000	2000
Total	10.9	2,472	1,880	2,940	447	1,246	2,003	1,123
Average					1,530.03			
Chromosome 1	1.7	411						
Chromosome 2	1.7	407						
Chromosome 3	1.5	333						
Chromosome 4	1.4	308						
Chromosome 5	1.3	308						
Chromosome 6	1.1	276						
Chromosome 7	0.9	209						
Chromosome 8	0.9	211						
Mitochondrion	0.4	9						

#### Overview of transcriptional data by RNA-seq

After sequencing on the HiSeq 2000 system (Illumina), 1.44 Gb of raw reads were obtained by RNA-seq from all three samples, subsequently, and 35.55 million cleans reads were acquired after removing the adaptors and the low-quality reads, which accounted for 98% of the total reads (Additional file 1: Table S1). The clean reads were aligned to the reference genome and genes using the SOAP2 software, with a tolerance of no more than 2 bp mismatches. The number of reads that uniquely mapped the reference genome and genes accounted for ~74–89% and ~35–57%, respectively (Additional file 1: Table S1).

### Quantification analysis of gene expression from *K. marxianus* Y179

#### Overall differential gene expression and validation by qPCR

**Fig. 2:**
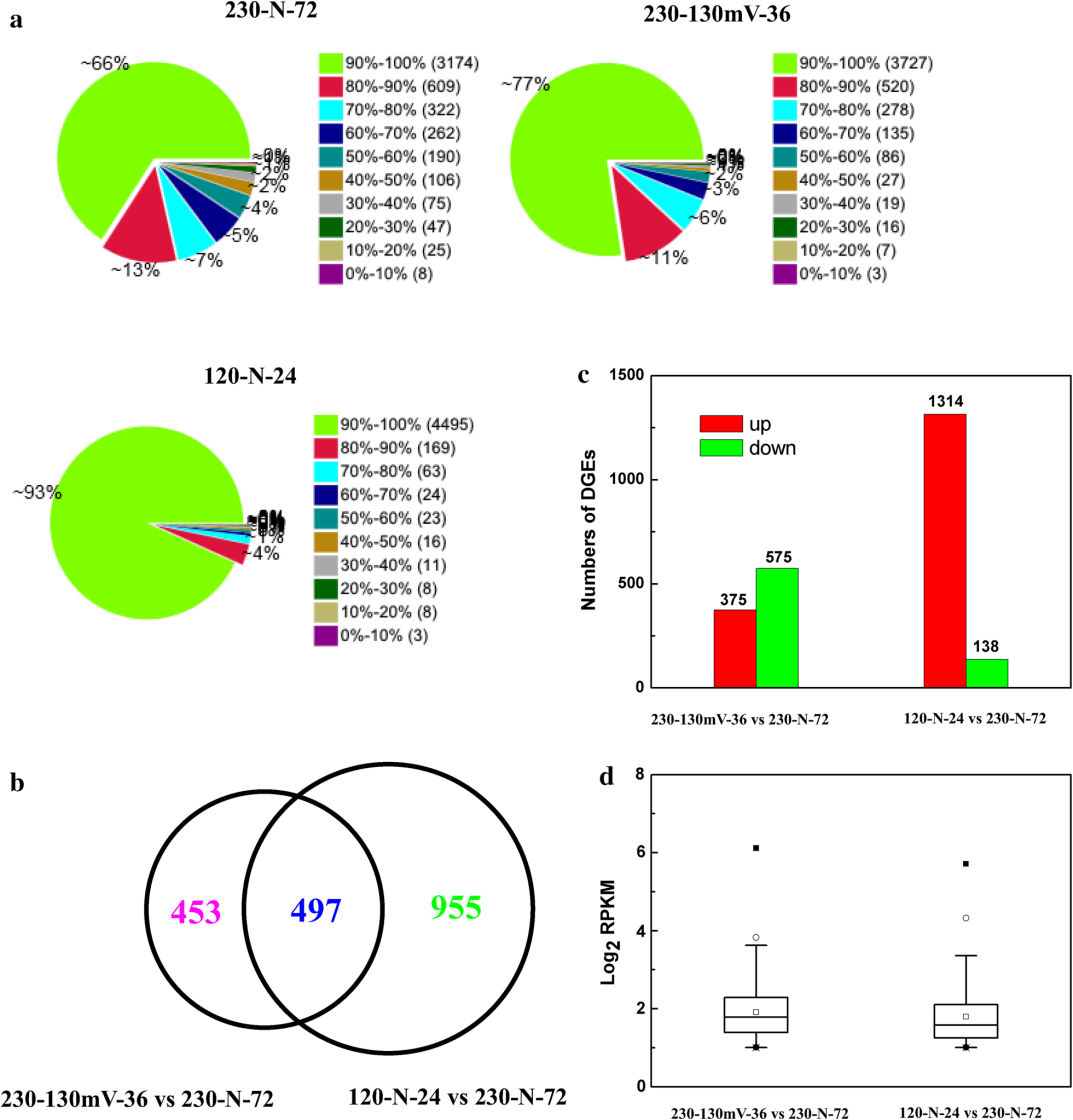
Overview of differentially expressed genes from RNA-seq. **a** Distribution of the genes’ coverage. **b** The overlap of differentially expressed genes between 230-130mV-36 vs 230-N-72 and 120-N-24 vs 230-N-72; **c** overview of up-regulated and down-regulated gene expression between 230-130mV-36 vs 230-N-72 and 120-N-24 vs 230-N-72; **d** box plot of log2 RPKM for comparison between 230-130mV-36 vs 230-N-72 and 120-N-24 vs 230-N-72 (*P* ≤ 0.001, log2 RPKM ≥1).

The levels of gene expression, normalized as reads per kilobase per million mapped reads (RPKM), were applied to the fold changes of the differentially expressed genes (DEGs). The annotated genes were able to be covered by the clean reads from RNA-seq, ~66–93% of which achieved over a 90% coverage of the known genes (Fig. 2a).

Two comparison modules among the three samples, 230-130, mV-36 vs 230-N-72, and 120-N-24 vs 230-N-72, were performed to investigate the significantly differentially expressed genes between micro-aeration and hypoxic conditions, or between low and high inulin concentrations (Additional file 1: Figure S1). In the first module, of 1,840 DEGs, 950 genes had annotation information, including 696 genes from KEGG and 520 genes from GO, when setting the cutoff values of screening as FDR ≤0.001 and the absolute value of log2 ratio ≥1. In the other module, 1,452 of 2,658 DEGs were obtained with annotation information, including 1,095 genes from KEGG and 820 genes from GO. Thus, it could be seen that, compared with aeration and hypoxic conditions, a larger difference in gene expression between low and high inulin concentrations were observed during ethanol production by *K. marxianus* Y179.

A further statistical analysis of genes containing annotations in the two modules is illustrated in Fig. 2b–d, which showed that 497 mutual genes changed between two modules. Considering the up- or down-regulation of genes, it was remarkable that a great deal of genes were up-regulated (including genes related to ethanol metabolism, transcriptional factors, and so on) in the module of 120-N-24 vs 230-N-72, which might explain the inhibition of high inulin concentrations (Fig. 2c). The distribution of RPKM in 230-130mV-36 vs 230-N-72 was slightly higher than that of 120-N-24 vs 230-N-72, which might be due to the fewer DEGs in the module of 230-130mV-36 vs 230-N-72.

Six genes involved in various pathways were selected to conduct the qPCR experiments to validate the reliability of data from RNA-seq. As illustrated in Table 3, though there were a few differences between the fold changes of RNA-seq and qPCR, the trends of up- or down-regulation of all six genes chosen were the same, which consequently demonstrated the accuracy of the trends of gene expression change obtained by RNA-seq.

**Table 3:** Comparison of change folds in DEGs between RNA-seq and qPCR

No.	Gene	Gene ID	RNA-seq		qPCR	
			230-130mV-36/230-N-72	120-N-24/230-N-72	230-130mV-36/230-N-72	120-N-24/230-N-72
1	*INU1*	allA4270	2.21	3.85	1.52	1.81
2	*FBP1*	allA1368	1.20	69.32	0.40	37.25
3	*HXK1*	allA0887	4.57	8.24	4.84	4.28
4	*CTT1*	allA1698	19.18	4.01	9.43	1.74
5	*GPM1*	allA4044	0.27	0.14	0.09	0.04
6	*GDP1*	allA4645	0.06	0.96	0.05	0.40

#### Characterization of DEGs involved in central carbon metabolism

**Fig. 3:**
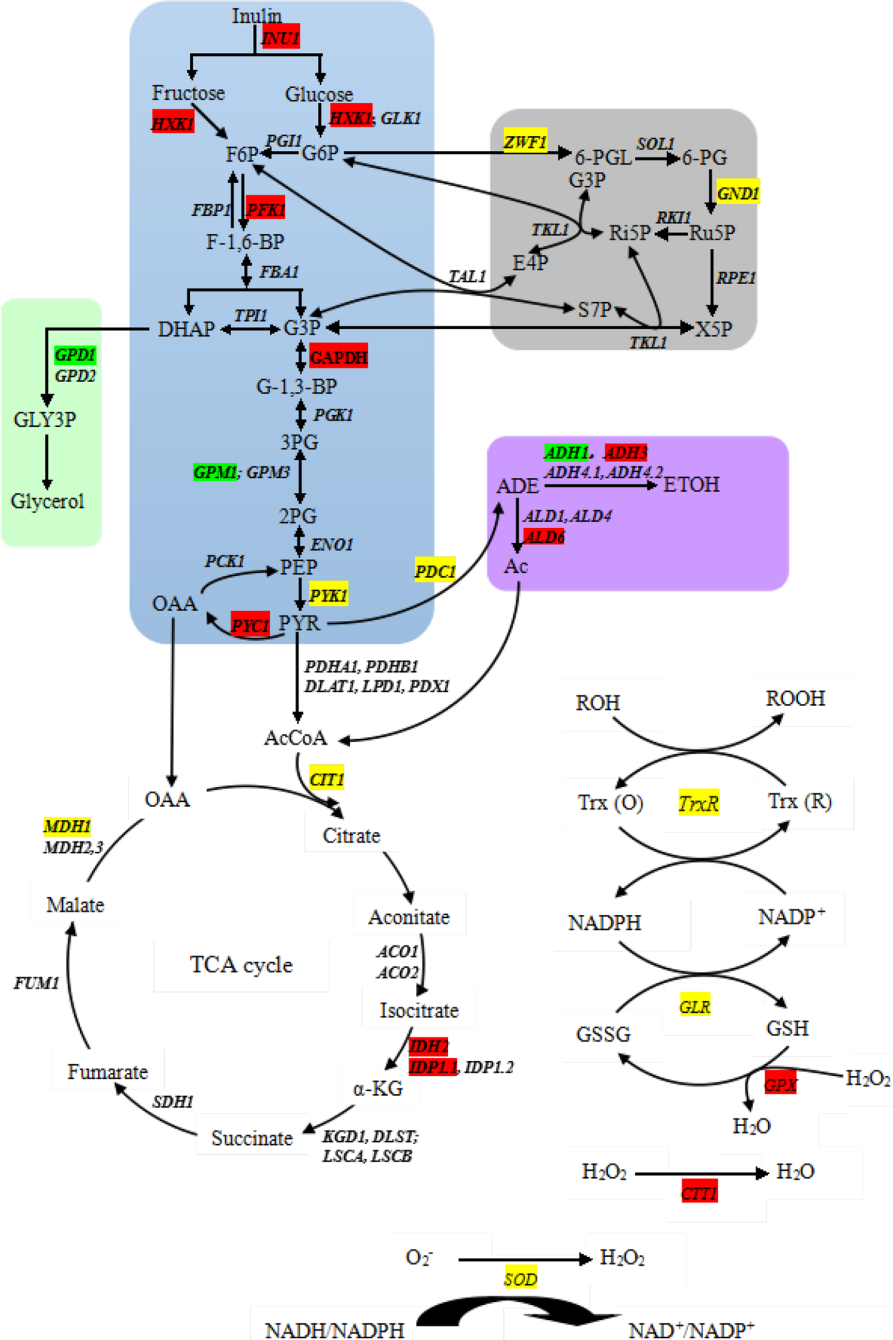
Profiles of central and oxidative stress response metabolism pathways of *K. marxianus*. The network included glycolysis (EMP), the ethanol and glycerol formation pathway, the TCA cycle, the pentose phosphate pathway (PPP), and the oxidative stress response pathway. Genes marked in *red*, *green*, and *yellow* represented up-regulated, down-regulated, or a mixture of up-regulated and down-regulated RPKMs, respectively, in 230-N-72, 230-130mV-36 and 120-N-24 conditions. Further details are given in Table 5.

**Fig. 4:**
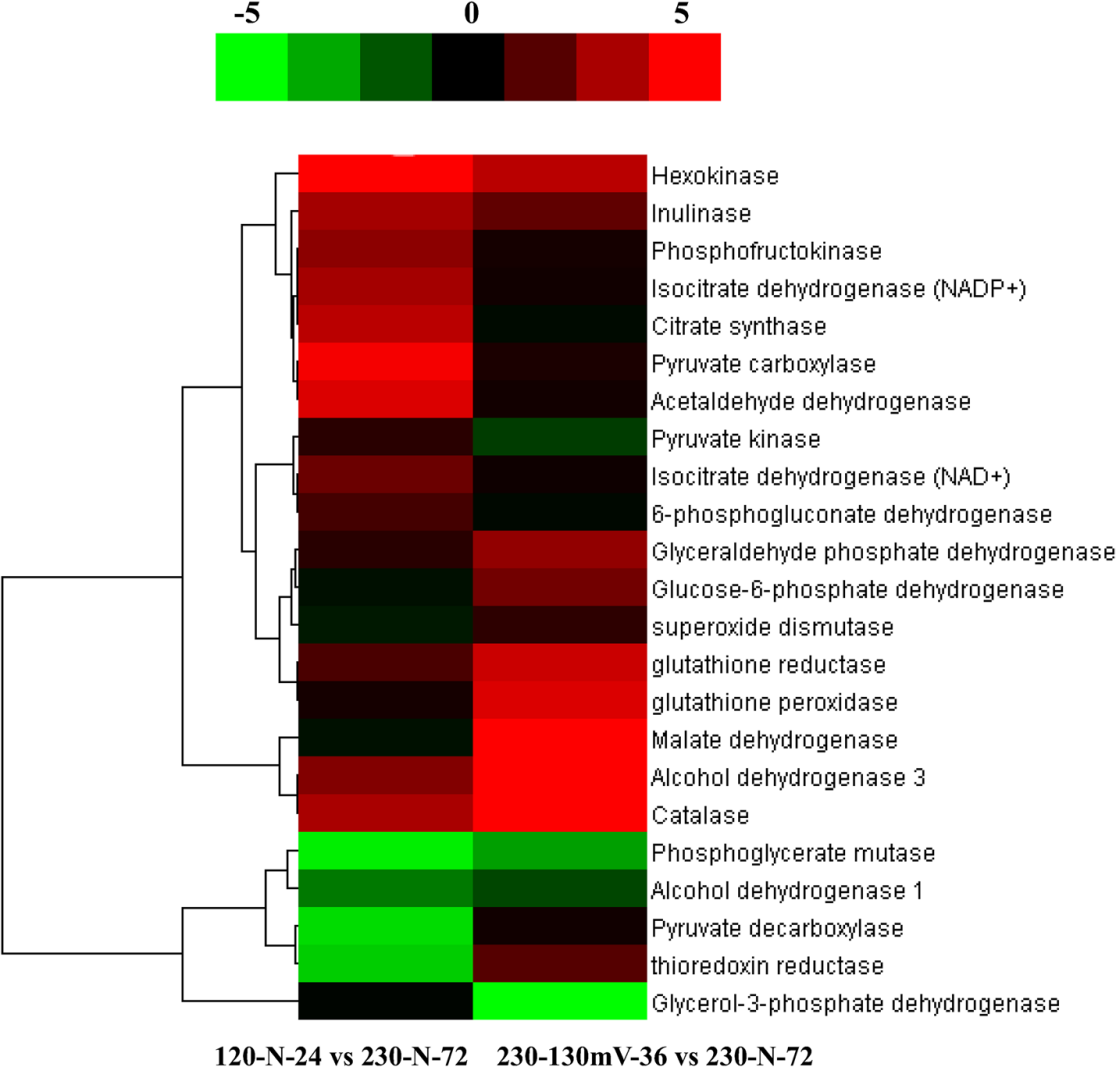
Cluster analyses of key genes from central and oxidative stress response metabolism pathways. Differentially expressed genes were clustered by log2-based expression values; the *rows* indicate different genes and the *columns* indicate two comparisons. *Green*, *black*, and *red* indicate up-regulated, unchanged, and down-regulated genes, respectively.

Of 1,880 genes containing KEGG annotations in *K. marxianus* Y179, more than 88 KEGG metabolic pathways were categorized; 35 and 56 DEGs (fold-change ≥2) were related to the central carbon metabolic pathways (glycolysis/gluconeogenesis, TCA cycle, and PPP) or 230-130mV-36 vs 230-N-72 and 120-N-24 vs 230-N-72, respectively. According to these KEGG pathways, we reconstructed the metabolic network containing the central carbon metabolic pathway, ethanol and glycerol formation pathways, and the related intermediate enzymes (Figs. 3, 4).

Inulinase encoded by *INU1* in *K. marxianus* Y179 might, to a large extent, affect the sugar consumption rates in ethanol fermentation. Compared with 230-N-72, micro-aeration (230-130mV-36) and low inulin concentration (120-N-24) up-regulated *INU1* by 1.14- and 1.94-fold, respectively, which was in accord with the inulinase activities of the supernatants (Table 1) and the previously published results [19, 20]. Hexokinase and glucokinase, encoded by *HXK1* and *GLK1*, respectively, which are crucial enzymes for controlling glucose and fructose in the central carbon metabolic pathway, were overexpressed by 2.2- and 3.0-fold under micro-aerobic and low inulin conditions in our systems, respectively. The overexpression of these three genes might explain the phenomenon of fast inulin consumption rates.

Phosphoglycerate mutase (encoded by *GPM1*) catalyzed the transition between 2-phosphoglycerate and 3-phosphoglycerate. Three kinds of isozymes were found in *S. cerevisiae*, encoded by *GPM1*, *GPM2*, and *GPM3* [21, 22]. Gpm1p and Gpm3p were detected in *K. marxianus* Y179, the identities of which were 87 and 57%, respectively, in contrast to that in *S. cerevisiae*. The expression of *GPM1* under aerobic and low inulin conditions was down-regulated by 1.89- and 2.83-fold, respectively. Papini et al. [23] also made it clear that the *GPM1* of *S. cerevisiae* were substantially up-regulated when using ethanol as the only carbon source. Consequently, the differential expression of *GPM1* in *K. marxianus* Y179 was inferred to be associated with respiratory growth on non-fermentable substrates, like ethanol, to maintain the energy balance in the central carbon metabolic pathway. To enhance a high ethanol yield, silencing *GPM1* in *K. marxianus* Y179 is an apparent alternative option to decrease the loss of ethanol as the substrate.

Pyruvate is the pivotal intermediate metabolite of the entire central carbon metabolism and determines the carbon fluxes to the TCA cycle or to ethanol formation. Pyruvate kinase, which catalyzes phosphoenolpyruvate into pyruvate, is regarded as the rate-limiting enzyme. Two kinds of pyruvate kinases, Pyk1p and Pyk2p (encoded by *CDC19* and *PYK2*, respectively), have been observed in *S. cerevisiae* so far [24, 25]. It was indicated previously that the expression of Pyk1p had nothing to do with the accumulation of ethanol [26], but it might be concerned with glucose utilization under the conditions of high temperature or resistance to environmental stress [24]. Only one kind of pyruvate kinase was detected in *K. marxianus* Y179, KmPyk1p, the identity of which was 86% homologous with Pyk1p in *S. cerevisiae*. However, the expressions of *KmPYK1* under three conditions demonstrated few differences (Additional file 1: Table S2).

To our surprise, the expression of malate dehydrogenase (Mdh1p) in the sample of 230-130mV-36 increased by up to 4.6-fold. The enhanced transcription of mdh1p, a key enzyme in the TCA cycle, might be speculated to have other biological functions, because there was no obvious enhancement of the TCA cycle in 230-130mV-36. Previous results showed that *MDH* could be regarded as a transhydrogenase-like shunt, which regulated the redox state in *S. cerevisiae* cells [27]. What is more important, research from Lushchak et al. [28] indicated that the expression level of Mdh1p in SOD-defective *S. cerevisiae* was up-regulated to work as a protective mechanism under oxidative stress. Consequently, the up-regulation of *KmMDH1* in 230-130mV-36 might have something to do with this. On the other hand, PPP provided and balanced NADP^+^/NADPH or NAD^+^/NADH ratio in cells, so the expression of genes involved in PPP were promoted under aerobic conditions (230-130mV-36) for the regulation and balance of the redox state in the cell (Figs. 3, 4).

#### Characterization of DEGs related to ethanol, glycerol, and by-products formation

Pyruvate was first converted to acetaldehyde by pyruvate decarboxylase (pdc), which was then reduced to ethanol by alcohol dehydrogenase (adh) in the pathway of ethanol formation. The pyruvate decarboxylase, *PDC1*, played a crucial role in the decarboxylation of pyruvate in cells, and the expression of *PDC1* in high ethanol concentrations (230-N-72 and 230-130mV-36 against 120-N-24) was enhanced by roughly threefold, which agreed with previous results [26]. In addition, an appropriate aeration resulted in a slightly higher RPKM value of *PDC1* (25,820 vs 22,334). As mentioned by Suleau et al. [5], the up-regulation of *PDC1* ensured carbon fluxing to the fermentative pathways rather than the TCA cycle, which indicated the relatively ideal results of micro-aeration by controlling ORP at −130 mV.

**Fig. 5:**
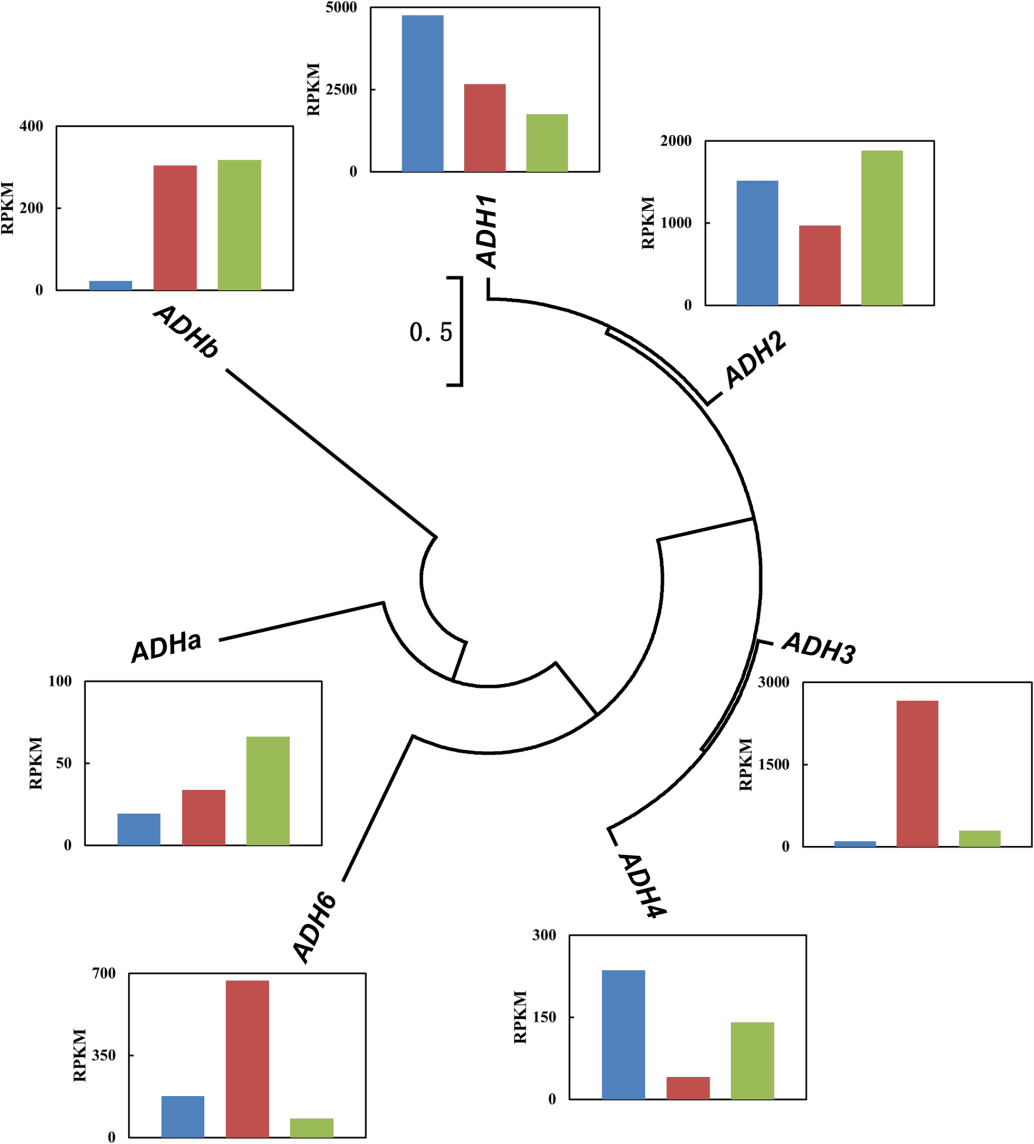
Phylogenetic tree of predicted alcohol dehydrogenase genes in Y179 with transcript abundance under three conditions. The neighbor-joining algorithm was used to construct the tree with protein sequences of several alcohol dehydrogenases. The transcript abundance of each *ADH* gene was indicated by the RPKM values shown in Additional file 1: Table S2, and each *column* in the figures represent the expression of *ADH* genes under certain conditions: *blue* 230-N-72; *red* 230-130mV-36; and *green* 120-N-24.

Acetaldehyde is reversibly reduced to ethanol by alcohol dehydrogenase, which is a vital enzyme for the metabolism of ethanol in yeasts. At least seven kinds of alcohol dehydrogenase were found in *S. cerevisiae*, Adh1p–Adh7p [29]. Seven alcohol dehydrogenases detected in *K. marxianus* Y179 were analyzed using a phylogenetic tree (Fig. 5). The levels of expression (RPKM values) demonstrated the remarkable functions of four *ADH* genes (Adh1p–Adh4p), which could be classified into two types according to the phylogenetic tree. Adh1p and Adh2p were found to be located in the cytosol, were the main functional alcohol dehydrogenases under anaerobic conditions, and kept a high transcriptional level under very high gravity conditions [26, 30], which was in accordance with our results in this article. Additionally, Adh3p and Adh4p were located in the mitochondria. Adh4p showed no structural or functional homologies with the other three enzymes [29], and its low RPKM value obtained in this study demonstrated a small role in ethanol metabolism in the presence of non-respiratory carbon sources, as described previously [31]. As reported previously, *ADH3*-disrupted *K. marxianus* was more sensitive to the reactive oxygen species [31]. The expression of *ADH3* in *K. marxianus* Y179 was dramatically up-regulated (over 10-fold) under aerobic conditions (230-130mV-36 against 230-N-72 and 120-N-24) (Fig. 5; Additional file 1: Table S2), which hence participated in the enhancement of respiration and played a significant role in the reduction reactions.

Glycerol, as a protective mechanism responding to environmental stress, played a key role in keeping high cell viabilities during ethanol fermentation. Two homologies of glycerol-3-phosphate dehydrogenase, key enzymes in glycerol metabolism, were discovered in *S. cerevisiae* (Gpd1p and Gpd2p), which exhibited an enhanced expression under high osmolarity and anaerobic conditions, respectively [32–34]. Our results revealed that there were also two kinds of glycerol-3-phosphate dehydrogenase in *K. marxianus* Y179, KmGpd1p and KmGpd2p. *GPD1* from *S. cerevisiae* was reported to be significantly down-regulated, due to a lack of dissolved oxygen [26]; however, in our study, the *KmGPD1* gene was apparently up-regulated under high inulin concentrations and anaerobic conditions (Additional file 1: Table S2), which agreed with the concentration obtained by HPLC in the supernatant (data not shown).

### Characterization of DEGs response to oxidative stress

Our previous fermentation indicated the delay of the process under relatively anoxic conditions and in turn an appropriate aeration volume promoted cell growth and ethanol production. From yet another angle, aeration resulted in the production of toxic compounds, called reactive oxygen species (ROS), including superoxide, peroxide, hydroxyl radical, and so on [35, 36]. As a consequence, a thorough protective mechanism exists in *K. marxianus* Y179 cells to protect it from damage by ROS.

The Trx-TrxR system is widely present in most yeast mitochondria and acts as a defense against ROS damages. Under aerobic conditions, the *TRXR* gene increased by 1.0-fold (230-N-72) and 3.4-fold (120-N-24), respectively. Glutathione reductase achieved the transition between oxidized and reduced glutathione, which was up-regulated by 1.5- to 2.4-fold under aerobic conditions. Peroxidase consumed ROS like O_2_^2−^ via the reduction of H_2_O_2_, which mainly includes catalase and glutathione peroxidase, and aeration enhanced them by 2.2- to 4.3-fold (230-N-72) and 2.3- to 2.6-fold (120-N-24), respectively. Cu–Zn superoxide dismutase was also up-regulated by roughly 1.0-fold under aerobic conditions to remove the superoxide anion in cells. Blanco et al. [35] reported the responses of gene transcriptional levels in *K. lactis* to hypoxic and oxidative stress, and they found that the *TRXR* and *CTT1* genes in wild-type *K. lactis* were obviously up-regulated during the aerobiosis–hypoxia shift. In spite of ROS being generated by aeration (230-130mV-36), the obvious up-regulation of various genes related to oxidative stresses removed most ROS damages and guaranteed relatively high cell viabilities to achieve an ideal result.

A type of alkyl hydroperoxide reductase (encoded by *KmallA2475*) was worthy of attention because of its high transcriptional level (RPKM up to 20,000), which increased by 1.3- to 3.3-fold compared to that under anoxic conditions. On the basis of sequence analysis, it belonged to one of the five types of peroxiredoxin (Tas1p) [37], which occupied the highest concentration in cells [38]. The homologous identity of *KmallA2475* with that in *S. cerevisiae* was up to 90%, and it could be predicted that *KmallA2475* in *K. marxianus* Y179 might be one of the most important enzymes for defense against ROS damages.

In addition, the high transcriptional level of a polyamine transporter (encoded by *TPO1*) piqued our interests (Additional file 1: Table S2). Endogenous polyamine protects cells from damage by some fermentation inhibitors and ethanol by binding to plasma membranes, ribosomes, and DNA [39]. Aranda et al. [40] found that the addition of acetaldehyde up-regulated *TPO1* in *S. cerevisiae*. Moreover, a *TPO1*-defective strain showed an enhanced tolerance of fermentation inhibitors, such as furfural and acetic acid [41]. However, the aerobic conditions increased the *TPO1* gene expression by 2.6- to 3.7-fold accordingly in our study. Though the specific underlying reasons and mechanisms are still unclear, we predict that this may be related to the acetaldehyde content in ethanol metabolism and that it may belong to a kind of protective mechanism for cells, especially for defending against damages by ROS from aeration by transporting a special type of polyamine into cells.

### Characterization of DEGs associated with sugar transporters

Considering the homology between *K. marxianus* and *K. lactis*, we aligned the predicted hexose transporter genes in *K. marxianus* Y179 to the known transporters in *K. lactis* and obtained a similar transport system (Table 4). *K. lactis* embraced a relatively simple transport system and related genes [42]. The transport of hexoses by *K. lactis* mainly relied on two transporters: a low-affinity glucose transporter, rag1p [43], and a high-affinity glucose transporter, hgt1p [44]. In most *K. lactis* cells, rag1p was usually replaced by two high homologous proteins, Kht1p and Kht2p [42]. In addition, *K. lactis* also expressed the *FRT1* gene, which encoded a hexose transporter with a high affinity with fructose, which contributed to parts of hexose transport [45].

**Table 4:** Hexose transporters in *K. lactis* and *K. marxianus*

Transporter in *KL*	Functions	Gene in *KM*	Identities (%)	RPKM		
				230-N-72	230-130mV-36	120-N-24
*RAG1/KHT1*	Low-affinity glucose transporter	*KMallA1640*	79	243.57	149.56	629.7
		*KMallA1641*	78	337.99	27.69	112.28
*KHT2*		*KMallA1637*	83	111.28	65.63	138.4
		*KMallA1638*	82	238.77	126.54	317.6
		*KMallA1639*	80	127.34	282.86	350.28
*HGT1*	High-affinity glucose transporter	*KMallA4236*	80	111.48	62.78	762.2
*FRT1*	High-affinity fructose transporter	*KMallA0822*	79	194.02	103.62	745.55

Rag1p/kht1p in *K. marxianus* Y179 was induced by high concentrations of glucose or fructose, and we detected two predicted open reading frames (ORFs) that encoded the protein (*KmallA1640* and *KmallA1641*). Kht2p, encoded by three ORFs (*KmallA1637*, *KmallA1638*, and *KmallA1639*), was up-regulated under low glucose concentrations. The up-regulation of two high-affinity transporters, Hgt1p and Frt1p, in the sample of 120-N-24 might account for the low residual sugar concentration. Therefore, we speculated that if the expression of these transporters were increased at the first stage of fermentation, the process of ethanol production might be accelerated to some extent.

### Characterization of DEGs referring to transcriptional regulations

Transcriptional regulation may be associated with transcription factors (TFs). The phenomenon of glucose repression exists widely in yeast cells, where mig1p, encoded by *MIG1*, may be a crucial TF. The *MIG1* gene had been detected in various yeasts, including *S. cerevisiae*, *K. lactis*, and *K. marxianus* [46, 47]. Cassart et al. [47] demonstrated that the expression of *MIG1* was regulated by glucose in the media, and the promoters of invertase (encoded by *SUC2*) and inulinase (encoded by *INU1*) were repressed by Mig1p in the presence of glucose. In our experiments, the enhanced expression of *KmMIG1* was observed under high inulin and anoxic conditions (RPKM = 1,308, Additional file 1: Table S2). After the employment of low inulin and aerobic conditions, the expression of *KmMIG1* was down-regulated by 1.4- to 2.5-fold. Consequently, an appropriate manipulation of the *MIG1* gene (for example, lowering or silencing its expression at the initial stage of fermentation) may improve some key enzymes’ activities, like inulinase in the process of ethanol production from inulin, thus fundamentally solving the complex problems.

In contrast to the behavior of *MIG1*, the *CCR4/POP2* regulation system contributes to glucose derepression. Ccr4p is a complex that regulates the expression of various genes in yeast cells. Known to be part of the Ccr4p complex [48, 49], similar to that of Pop2p, Ccr4p can resist glucose repression with the assistance of glucose-repressible alcohol dehydrogenase (encoded by *AHD2*) [49]. The relatively low transcriptional levels of the two TFs in *K. marxianus* Y179 (*KmPOP2* and *KmCCR4*), which were homologous to those in *S. cerevisiae*, indicated that the *CCR4/POP2* system did little to regulate gene expression in *K. marxianus* Y179. The inconspicuous differences in the expression of *ADH2* among three samples had also proven the above conclusion (Fig. 5; Additional file 1: Table S2).

On the other hand, the expression of some functional proteins will also affect the gene expression and the maintenance of their activities. The heat shock protein (*HSP*) family is a family of chaperones that assist proteins to fold correctly and maintain activities under some strict environmental stresses. Hsp26 had a very high transcriptional level in *K. marxianus* Y179 during ethanol fermentation, up to 53% compared to that in *S. cerevisiae*, which was one of the ten highest expressed genes in all three samples (Additional file 1: Table S2). Amorós et al. [50] discovered that *HSP26* in *S. cerevisiae* was up-regulated under carbon source starvation and oxidative and osmotic stresses. As a consequence, it can be inferred that the enhanced expression of *KmHSP26* in *K. marxianus* Y179 may be related to the low inulin and high ethanol conditions at the final stage of fermentation.

Hsp42 was also previously reported in *S. cerevisiae* under ethanol fermentation conditions [26]. The expression profile of Hsp42 was also found to be correlated with an increase in ethanol concentrations, and its expression was induced by high ethanol concentrations, which was consistent with our results in this article. A 1.0-fold up-regulation of *KmHSP42* expression was observed under high ethanol and anoxic conditions (Additional file 1: Table S2), which might be conducive to conserving the enzyme activities of cells under very high ethanol concentrations. Hsp31 was induced by increasing oxidative stress in *S. cerevisiae* [51]. In accordance with that, we found that *KmHSP31* was up-regulated by 2.7- to 3.6-fold under aerobic conditions (230-130mV-36) in contrast to those in anoxic conditions, which might reduce the damages of enzymes in cells under high oxidative stress.

### Characterization of DEGs related to autophagy

**Fig. 6:**
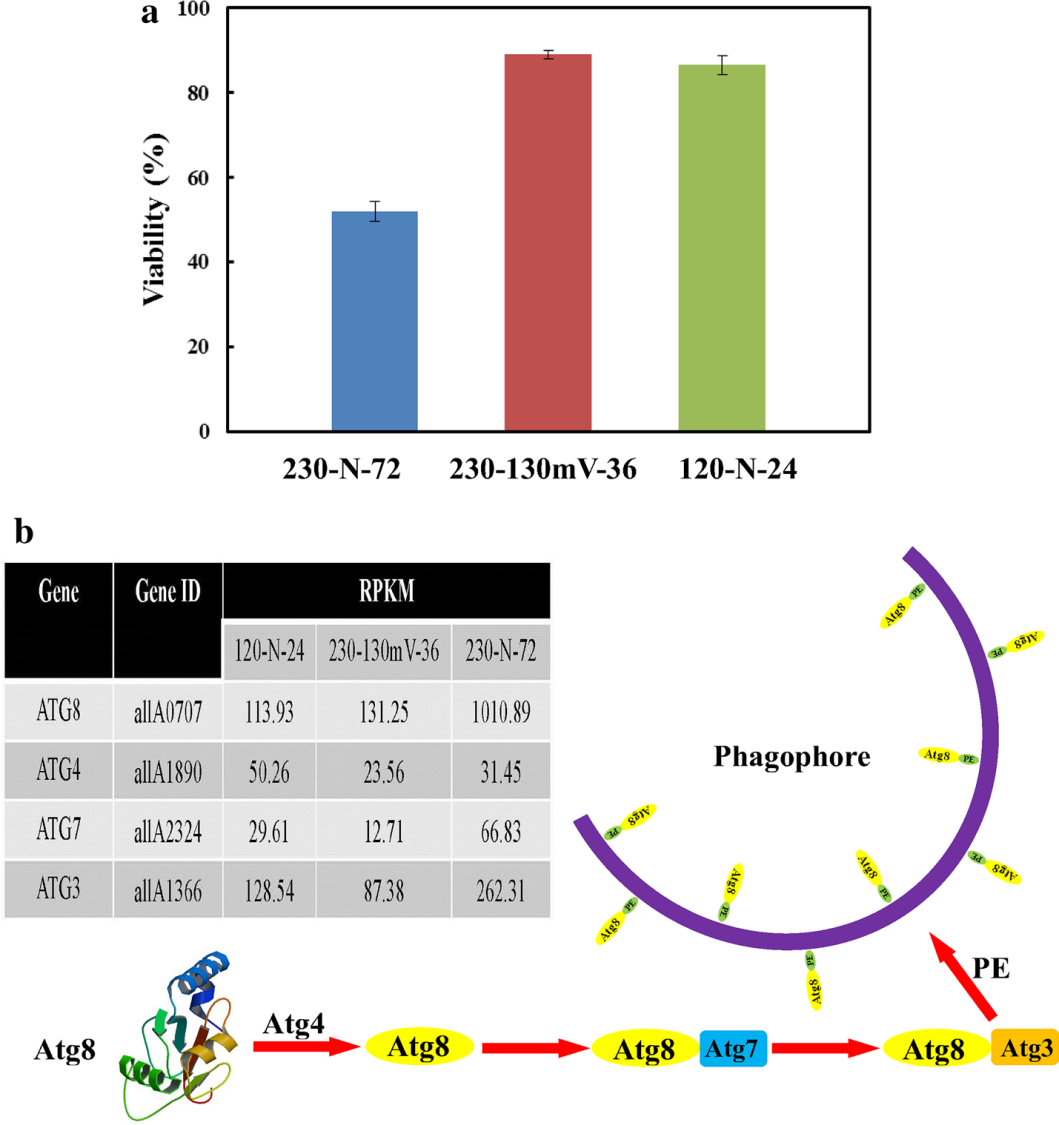
Profile of gene expression related to cell viability and non-selective autophagy in *K. marxianus* Y179. **a** Cell viabilities of three conditions at their own fermentative terminal. **b** Transcript abundance of *ATG* genes related to non-selective autophagy in *K. marxianus* Y179 and a brief introduction to the mechanism of non-selective autophagy.

Autophagy is supposed to be a conserved catabolic process in eukaryotes to maintain a steady state by the degradation of damaged cellular organelles and other dysfunctional macromolecules, like proteins [52]. Autophagy has been proven to make great differences and to guarantee the survival of yeasts under various stressful conditions [53]. There were great differences observed between the viability of cells under three conditions (Fig. 6a), which might result in different fermentative profiles. It was inferred that cell viabilities were greatly related to the fermentation time and the residual sugar concentration at the end. Consequently, having a better understanding of DEGs related to autophagy may provide references for optimizing the fermentative process.

It was detected that non-selective autophagy played a vital role for maintaining the cell survival in the systems of ethanol production from inulin by *K. marxianus* Y179 (Fig. 6). Non-selective autophagy, also called macroautophagy, engulfs the cytoplasm to form autophagosomes, which comprise the main degradation process in eukaryotes [54]. Three DEGs related to autophagy, *ATG3*, *ATG7*, and *ATG8* (Fig. 6b; Table 5), were identified to contribute to the formation of autophagosomes [55]. Atg8, a ubiquitin-like protein, was up-regulated by almost 3.0-fold under the condition of 230-N-72, which was in line with the results of cell viabilities (Fig. 6a). Moreover, it was reported that when macroautophagy occurred in cells, the expression of *ATG8* would greatly increase to demonstrate a positive correlation with the size of autophagosomes [55, 56]. Hence, the expression of *ATG* genes might be involved in the regulation of viabilities in the process of ethanol production by *K. marxianus* Y179.

**Table 5:** General review of autophagy-related genes (*ATG*) in *K. marxianus*

Gene	Gene ID	Size (bp)	RPKM			Source
			120-N-24h	230-130mV-36h	230-N-72h	
ATG1	allA2788	2,511	251.63	168.95	164.18	This study
ATG2	allA1909	4,500	53.51	32.78	34.03	This study
ATG3	allA1366	927	128.54	87.38	262.31	This study
ATG4	allA1890	1,356	50.26	23.56	31.45	This study
ATG5	allA3366	816	20.84	3.60	2.45	This study
ATG6	allA1725	1,401	36.51	6.98	15.84	This study
ATG7	allA2324	1,821	29.61	12.71	66.83	This study
ATG8	allA0707	375	113.93	131.25	1,010.89	This study
ATG9	allA0163	2,721	34.98	6.23	11.46	This study
ATG10	KLMA_80409	444	BAO42720.1^a^			
ATG11	allA0263	3,255	85.22	11.92	34.94	This study
ATG12	None^b^					
ATG13	allA2363	2,139	97.01	188.12	249.63	This study
ATG14	KLMA_20709	918	BAO39167.1			
ATG15	allA2983	1,635	128.96	96.59	51.56	This study
ATG16	allA3834	381	21.74	20.96	21.60	This study
ATG17	allA4154	1,266	19.40	10.68	8.08	This study
ATG18	KLMA_20474	1,542	BAO38932.1			
ATG19	allA2869	1,743	76.03	36.47	64.82	This study
ATG20	allA0816	1,803	113.96	110.55	640.71	This study
ATG21	allA4285	1,185	75.91	34.11	20.63	This study
ATG22	KLMA_10053	1,491	BAO37675.1			
ATG23	allA1449	1,329	49.09	21.95	74.88	This study
ATG24/SNX4	allA2880	1,194	46.00	13.79	23.39	This study
ATG25	None					
ATG26	allA4736	3,696	432.33	104.68	251.49	This study
ATG27	allA4445	840	60.38	29.88	140.73	This study
ATG28	None					
ATG29	KLMA_30451	489	BAO39746.1			
ATG30	None					
ATG31	None					
ATG32	allA3211	1,542	87.64	24.42	135.53	This study
ATG33	None					
ATG34	None					
ATG35	None					
ATG36	None					

^a^Accession number of protein sequences in NCBI.

^b^No related sequences reported in *K. marxianus*.

## Conclusions

A global transcriptome analysis of the non-conventional yeast, *K. marxianus* Y179, was conducted for ethanol production under various fermentative conditions. Hundreds of differentially expressed genes associated with the whole ethanol metabolism network were identified. Despite being reported as the “crabtree-negative” species, *K. marxianus* Y179 could achieve favorable fermentative profiles with an appropriate aeration volume, which had been verified by its transcriptional response. Genes related to oxidative stress and autophagy were apparently up-regulated or down-regulated to guarantee high cell viabilities, which were supposed to be the primary reasons of the ideal result. Moreover, the release of glucose repression and increased inulinase activity at the initial stage of fermentation, by controlling the transcription factor Mig1, may help to improve productivity. Consequently, information on the genome annotation and transcriptome data provided a valuable reference for future studies on the optimization and reconstruction of the metabolism network in *K. marxianus.*

## Methods

### Strains, media, and culture conditions

*Kluyveromyces marxianus* Y179 was deposited at the China Center for Type Culture Collection (CCTCC) with a reference number of M202031. The strain was maintained on a YPD agar slant composed of 20 g/L glucose, 20 g/L peptone, and 10 g/L yeast extract at 4°C for routine use. For long-term preservation, cultures were stored at −80°C in 20% glycerol.

### Genome annotation

The genome information of *K. marxianus* was downloaded from the NCBI database (http://www.ncbi.nlm.nih.gov/Taxonomy/Browser/wwwtax.cgi?id=1162310) [8]. The whole-genome sequences above were annotated according to the Gene Ontology (GO) database and Kyoto Encyclopedia of Genes and Genomes (KEGG) database to obtain the reference genome and annotated gene information.

### Transcriptome analysis of *K. marxianus* Y179

#### Cell growth conditions

Yeast cells that were cultivated in 100 mL of YPD medium in 250-mL flasks for 16–18 h were inoculated into a 3-L fermenter, with a 1.0-L working volume, at 30°C and 150 rpm. To study the transcriptional differences at high concentrations of substrate and aeration, three fermentation conditions were carried out: 230 g/L inulin in YP medium (20 g/L peptone, 10 g/L yeast extract) without aeration (230-N-72), 230 g/L inulin in YP medium controlling the redox potential at −130 mV (230-130mV-36), and 120 g/L inulin in YP medium without aeration (120-N-24). The ORP throughout the process was controlled by a sterile ORP electrode and a PID (Proportional, Integral, and Derivative) control algorithm, as described previously [26].

#### RNA isolation

Samples were taken after fermentation, under three conditions, at 72, 36, and 24 h, respectively. Cell pellets were collected by centrifugation at 5,000*g* at 4°C for 5 min, and were then frozen by liquid nitrogen. The total RNA of every sample was extracted by the RNeasy^®^ Mini Kit (Qiagen, Hilden, Germany) according to the manufacturer’s instructions. The 2130 Bioanalyzer (Agilent Technologies, Santa Clara, CA, USA) was used to determine the RNA quality and integrity, and the RNA integrity number (RIN) of each sample used for RNA-seq was no less than 6.5.

#### Preparation of cDNA library and sequencing

The cDNA libraries were constructed and sequenced at the Beijing Genomics Institute (BGI, Shenzhen, China). The final cDNA libraries were qualified and quantified using the 2130 Bioanaylzer (Agilent Technologies) and the StepOnePlus Real-Time PCR System (Applied Biosystems). Afterward, the library products were ready for sequencing via the HiSeq 2000 system (Illumina). To detect the differences of gene expressions among various samples, single-end technology was used to obtain about 40–50 bp reads in a single run.

#### Quantification analysis of gene expression by RNA-seq

To annotate and quantify the genes expressed in *K. marxianus* Y179, we first calculated the percentage of the gene covered by reads (gene coverage), which was determined as the ratio of the base number in a gene covered by unique mapping reads to the total base numbers of that gene. The levels of the differentially expressed genes (DEGs) were normalized by RPKM, defined as the number of reads per kilobase of the exon region per million mapped reads. Furthermore, in this article, the log2 ratio of RPKM between different samples was used to calculate the fold-change values. To screen the DEGs among various samples, we used a false discovery rate (FDR) of ≤0.001 and the absolute value of the log2 ratio ≥1 as the threshold to judge the significance of differential gene expression. More stringent criteria with a smaller FDR and a larger fold-change value can be used to identify DEGs.

#### Analysis of the DEGs of *K. marxianus* Y179 grown in different culture conditions

Expression pattern analysis of DEGs was clustered using Cluster software [57] and Java Treeview software [58]. The hierarchical clustering of the chosen experimental conditions and genes was carried out using the Euclidean distance as the formula of the distance matrix.

Gene Ontology (GO) enrichment analysis and functional classification helped to simplify huge amounts of data from RNA-seq and to identify the most significant DEGs from thousands of genes. To understand the distribution of gene functions of the species on the macro level, GO functional classification for DEGs was applied using the WEGO software [59] after obtaining the GO annotation of DEGs, as described above.

Kyoto Encyclopedia of Genes and Genomes was used as the major public pathway-related database to conduct the pathway enrichment analysis to further understand the biological functions of the genes. KEGG enrichment analysis identifies the significantly enriched metabolic pathways or the signal transduction pathways in DEGs, compared with the whole-genome background, of which the *Q* value was defined as no greater than 0.05.

A brief introduction and procedure of the transcriptome analysis of *K. marxianus* Y179 is illustrated in Additional file 1: Figure S1.

### RT-PCR and real-time quantitative PCR

RT-PCR and real-time quantitative PCR (qPCR) were performed to verify the accuracy of the gene expression levels obtained by RNA-seq. The same total RNA samples for RNA-seq were utilized to conduct the PCR reactions. The total RNA of each sample (1 µg) was reverse transcribed to cDNA using the PrimeScript^®^ RT reagent Kit (Takara Bio Inc.) according to the manufacturer’s protocol. Afterward, six differentially expressed genes involved in EMP, TCA, glycerol and ethanol formation, and stress response pathways were selected to perform qPCR, and the information of the primers is shown in Additional file 1: Table S3. A 10 times dilution of cDNA solution was used as the DNA template for the qPCR reaction and ddH_2_O was used as the negative control. A two-step PCR reaction was employed, and the system and conditions were as described in the manufacturer’s protocol of SYBR^®^*Premix Ex Taq*™ II (Takara Bio Inc.). Finally, the actin gene was selected as the endogenous reference gene, and the data analysis of the fold change of a specific gene was determined by the method of $2^{{ - \varDelta \varDelta C_{\text{T}} }}$ as described by Livak and Schmittgen [60]. Triplicate experiments were performed to guarantee the reproducibility of all the results.

### Analytical methods

#### Biomass, sugar, and ethanol determination

The cell concentration was measured using the optical density at 620 nm. Concentrations of the reducing sugar in inulin were determined by the dinitrosalicylic acid method [61], and the total sugar was firstly hydrolyzed by 0.2 M H_2_SO_4_ at 100°C for 1 h, then measured by the methods of Miller [61] after adding an equivalent amount of 0.4 M NaOH.

The ethanol was analyzed by gas chromatography (Agilent 6890A, Agilent Technologies) as previously described [19]. Briefly, a hydrogen flame ionization detector and isothermal capillary column (solid phase: cross-linked polyethylene glycol, carrier gas: nitrogen, and injector temperature: 250°C) were operated at 250 and 120°C, respectively.

#### Measurement of inulinase activity

Inulinase activity was measured according to the previous method [19, 20]. Briefly, 0.5 mL of culture supernatant was incubated with 2% (w/v) inulin prepared in 0.02 M sodium acetate buffer (pH 4.6) at 55°C for 10 min, and the reducing sugar was analyzed by the dinitrosalicylic acid method [61]. One enzyme unit was defined as the amount of fructose (μM) hydrolyzed per minute under the above conditions. Fructose was used as the standard substance to plot a standard curve.

#### Cell viability detection

The viabilities of the *K. marxianus* Y179 cells were roughly detected by the methylene blue stain technique [62].

Analysis of the biomass, sugars, ethanol, inulinase activity, and cell viabilities were done in duplication, and the mean values are shown in the results section.

## Additional file

Additional file 1:
**Figure S1.** Overview of transcriptomics analysis roadmap and procedure of RNA-seq analysis. **Table S1.** Summary of mapping results (mapping to genome and genes) from RNA-Seq. **Table S2.** Summary of DEGs involved in KEGG pathways. **Table S3.** Primes used in qPCR analysis.

## Abbreviations

CBP: consolidated bioprocessing
ORP: redox potential
DEG: differentially expressed gene
RNA-seq: next-generation sequencing technology
qPCR: real-time quantitative polymerase chain reaction
EMP: glycolysis
TCA: tricarboxylic acid cycle
PPP: pentose phosphate pathway
GO: Gene Ontology
KEGG: Kyoto Encyclopedia of Genes and Genomes
ROS: reactive oxygen species
