# Supplementary material for: Transcriptional analysis of Kluyveromyces marxianus for ethanol production from inulin using consolidated bioprocessing technology
Source: Biotechnol Biofuels. 2015 Aug 14;8:115. doi: 10.1186/s13068-015-0295-y (PMC4535673; doi:10.1186/s13068-015-0295-y)
Supplement: Additional file 1: — Figure S1. Overview of transcriptomics analysis roadmap and procedure of RNA-seq analysis. Table S1. Summary of mapping results (mapping to genome and genes) from RNA-Seq. Table S2. Summary of DEGs involved in KEGG pathways. Table S3. Primes used in qPCR analysis. [file 13068_2015_295_MOESM1_ESM.doc]

**Figure S1 Overview of transcriptomics analysis roadmap and procedure of RNA-seq analysis**

Table S1 Summary of mapping results (mapping to genome and genes) from RNA-Seq

| Sample ID | | 230-N-72 | 230-130mV-36 | 120-N-24 |
| --- | --- | --- | --- | --- |
| Total Reads | | 11527422 | 11928934 | 12092114 |
| Total BasePairs | | 576371100 | 596446700 | 604605700 |
| Total Mapped Reads | Genome | 9860674(85.54%) | 11116281(93.19%) | 9216469(76.22%) |
|  | Gene | 4057814(35.20%) | 6240161(52.31%) | 6958743(57.55%) |
| perfect match | Genome | 7899754(68.53) | 8869834(74.36%) | 7211058(59.63%) |
|  | Gene | 3175439(27.55%) | 4914812(41.20%) | 5519129(45.64%) |
| <=3bp mismatch | Genome | 1960920(17.01%) | 2246447(18.83%) | 2005411(16.58%) |
|  | Gene | 882375(7.65%) | 1325349(11.11%) | 1439614(11.91%) |
| unique match | Genome | 9662496(83.82%) | 10586075(88.74%) | 9003419(74.46%) |
|  | Gene | 4009682(34.78%) | 6135789(51.44%) | 6881569(56.91%) |

**Table S2** Summary of DEGs involved in KEGG pathways

| NO. | EC number | Enzyme | gene ID | Gene | Gene ORF size (bp) | RPKM | | | Pathway |
| --- | --- | --- | --- | --- | --- | --- | --- | --- | --- |
|  |  |  |  |  |  | 230-N-72h | 230-130mV-36h | 120-N-24h |  |
| 1 | 3.2.1.20 | Inulinase | allA4270 | *INU1* | 1671 | 1326.08 | 2924.44 | 5101.01 | EMP |
| 2 | 5.4.2.2 | Phosphoglucomutase | allA3255 | *PGM1* | 1701 | 386.48 | 561.66 | 1204.73 |  |
| 3 | 2.7.1.1 | Hexokinase | allA4774 | *GLK1* | 1446 | 177.30 | 246.16 | 552.42 |  |
|  |  |  | allA0887 | *HXK1* | 1458 | 434.65 | 1986.48 | 3583.06 |  |
| 4 | 5.1.3.15 | Glucose-6-phosphate 1-epimerase | allA3050 | *GPE1* | 891 | 59.34 | 1368.40 | 76.98 |  |
| 5 | 5.3.1.9 | Glucose-6-phosphate isomerase | allA4640 | *GPI1* | 1668 | 128.29 | 476.62 | 348.48 |  |
| 6 | 3.1.3.11 | Fructose-1,6-bisphosphatase I | allA1368 | *FBP1* | 1056 | 15.11 | 18.06 | 1047.48 |  |
| 7 | 2.7.1.11 | 6-phosphofructokinase | allA1359 | *PFK2* | 2817 | 102.17 | 121.73 | 322.10 |  |
|  |  |  | allA4481 | *PFK1* | 2937 | 384.07 | 607.35 | 424.37 |  |
| 8 | 1.2.1.12 | glyceraldehyde 3-phosphate dehydrogenase | allA0782 | *GAPDH1* | 996 | 1420.26 | 4706.24 | 1983.06 |  |
|  |  |  | allA3471 | *GAPDH2* | 1062 | 4.93 | 3.22 | 15.60 |  |
| 9 | 5.4.2.1 | Phosphoglycerate mutase | allA4642 | *GPM3* | 864 | 205.23 | 62.25 | 289.12 |  |
|  |  |  | allA4044 | *GPM1* | 744 | 33674.20 | 9112.32 | 4740.53 |  |
| 10 | 4.1.2.13 | fructose-bisphosphate aldolase | allA2277 | *FBA1* | 1086 | 2083.58 | 1383.51 | 2261.89 |  |
| 11 | 2.7.2.3 | phosphoglycerate kinase | allA4246 | *PGK1* | 1251 | 3306.74 | 2798.25 | 3102.28 |  |
| 12 | 4.2.1.11 | Enolase | allA4326 | *ENO1* | 1314 | 8527.3 | 7225.86 | 4786.35 |  |
| 13 | 5.3.1.1 | Triosephosphate isomerase | allA1997 | *TPI1* | 747 | 376.26 | 510.97 | 747.59 |  |
| 14 | 4.1.1.49 | Phosphoenolpyruvate carboxykinase | allA3218 | *PCK1* | 1632 | 294.32 | 142.21 | 593.11 |  |
| 15 | 2.7.1.40 | Pyruvate kinase | allA1086 | *PYK1* | 1506 | 1468.23 | 894.54 | 2070.51 |  |
| 16 | 4.1.1.1 | Pyruvate decarboxylase | allA1235 | *PDC1* | 1695 | 22334.58 | 25820.46 | 3677.82 | By-products formation pathway |
| 17 | 1.1.1.1 | Alcohol dehydrogenase | allA1971 | *ADH1* | 1047 | 4750.92 | 2672.25 | 1750.31 |  |
|  |  |  | allA2097 | *ADH2* | 1047 | 1516.15 | 969.93 | 1881.33 |  |
|  |  |  | allA0644 | *ADH3* | 1128 | 101.48 | 2670.65 | 294.37 |  |
|  |  |  | allA3324 | *ADH4* | 1170 | 236.18 | 41.09 | 140.97 |  |
|  |  |  | allA0627 | *ADH6* | 1101 | 177.14 | 670.27 | 81.83 |  |
|  |  |  | allA2529 | *ADHa* | 1161 | 19.33 | 33.69 | 66.34 |  |
|  |  |  | allA3486 | *ADHb* | 1257 | 22.42 | 304.30 | 317.22 |  |
| 18 | 1.2.1.3 | Aldehyde dehydrogenase | allA4815 | *ALD6* | 1524 | 615.96 | 717.36 | 3712.80 |  |
|  |  |  | allA3860 | *ALD1* | 1506 | 15.90 | 25.22 | 6.75 |  |
|  |  |  | allA1949 | *ALD4* | 1566 | 8.28 | 13.11 | 5.66 |  |
| 19 | 1.1.1.94 | Glycerol-3-phosphate dehydrogenase | allA4645 | *GPD1* | 1167 | 1029.43 | 65.78 | 988.07 |  |
|  | 1.1.5.3 |  | allA3222 | *GPD2* | 1929 | 16.81 | 48.24 | 227.80 |  |
| 20 | 1.2.4.1 | Pyruvate dehydrogenase (E1) | allA3339 | *PDA1* | 1242 | 87.55 | 60.10 | 150.46 | TCA |
|  |  |  | allA3452 | *PDB1* | 1062 | 58.24 | 106.04 | 200.60 |  |
| 21 | 2.3.1.12 | Dihydrolipoamide acetyltransferase（E2) | allA0788 | *PDX1* | 1251 | 25.92 | 38.82 | 106.17 |  |
|  |  |  | allA1025 |  | 1404 | 28.07 | 100.29 | 176.16 |  |
| 22 | 1.8.1.4 | Dihydrolipoamide dehydrogenase（E3) | allA0876 |  | 1482 | 72.19 | 261.40 | 279.36 |  |
| 23 | 6.2.1.1 | Acetyl-CoA synthetase | allA3093 |  | 2124 | 4.58 | 17.34 | 27.30 |  |
|  |  |  | allA1815 |  | 2055 | 348.43 | 215.24 | 1125.47 |  |
| 24 | 2.3.3.1 | Citrate synthase | allA3266 | *CIT1* | 1452 | 176.40 | 162.19 | 809.24 |  |
| 25 | 4.2.1.3 | Aconitate hydratase | allA2779 | *ACO1* | 2343 | 175.95 | 242.90 | 521.47 |  |
|  |  |  | allA4672 | *ACO2* | 2394 | 33.96 | 15.39 | 48.86 |  |
| 26 | 1.1.1.41 | Isocitrate dehydrogenase(NAD+) | allA3760 | *IDH2* | 1107 | 70.52 | 79.35 | 170.65 |  |
|  |  |  | allA0468 | *IDH1* | 1086 | 108.39 | 111.2 | 126.58 |  |
| 27 | 1.1.1.42 | Isocitrate dehydrogenase (NADP+) | allA2613 | *IDP1.1* | 1248 | 138.09 | 159.32 | 532.01 |  |
|  |  |  | allA2359 | *IDP1.2* | 1290 | 10.83 | 23.25 | 16.90 |  |
| 28 | 6.2.1.5 | Succinyl-CoA synthetase | allA3755 | *LSCA* | 957 | 206.21 | 189.20 | 142.29 |  |
|  |  |  | allA4485 | *LSCB* | 1275 | 72.57 | 108.78 | 154.89 |  |
| 29 | 2.1.3.61 | Dihydrolipoamide succinyltransferase (E2) | allA1775 |  | 1371 | 76.40 | 584.99 | 220.46 |  |
| 30 | 1.2.4.2 | 2-oxoglutarate dehydrogenase (E1) | allA0903 |  | 3063 | 44.38 | 70.29 | 174.21 |  |
| 31 | 6.4.1.1 | Pyruvate carboxylase | allA4568 | *PYC1* | 3528 | 31.95 | 40.01 | 238.32 |  |
| 32 | 4.2.1.2 | Fumarate hydratase | allA0564 |  | 1452 | 76.26 | 41.87 | 193.25 |  |
| 33 | 1.1.1.37 | Malate dehydrogenase | allA1133 | *MDH1* | 1017 | 810.48 | 17583.06 | 709.86 |  |
|  | 1.1.1.38 |  | allA2279 | *MDH2* | 1125 | 1387.53 | 239.32 | 767.40 |  |
|  |  |  | allA2504 | *MDH3* | 1500 | 464.21 | 145.59 | 922.17 |  |
| 34 | 1.1.1.39 | Malic enzyme | allA0965 | *MAE1* | 1929 | 55.98 | 71.82 | 270.14 |  |
| 35 | 2.3.3.9 | Malate synthase | allA1063 |  | 1668 | 227.42 | 71.03 | 202.38 |  |
| 36 | 1.1.1.49 | Glucose-6-phosphate 1-dehydrogenase | allA1907 | *ZWF1* | 1494 | 103.00 | 263.56 | 90.46 | PPP |
| 37 | 3.1.1.31 | 6-phosphogluconolactonase | allA0114 | *SOL1/CGR1* | 972 | 215.53 | 70.76 | 124.24 |  |
| 38 | 2.7.6.1 | Ribose-phosphate pyrophosphokinase | allA0979 |  | 963 | 93.49 | 35.03 | 250.49 |  |
|  |  |  | allA0698 |  | 969 | 359.81 | 145.82 | 141.27 |  |
|  |  |  | allA2420 |  | 1254 | 87.31 | 54.46 | 168.49 |  |
|  |  |  | allA4648 |  | 1482 | 95.59 | 71.04 | 186.30 |  |
| 39 | 2.2.1.1 | Transketolase | allA0639 | *TKL1* | 2040 | 138.51 | 219.70 | 415.50 |  |
| 40 | 5.1.3.1 | Ribulose-phosphate 3-epimerase | allA1293 | *RPE1* | 972 | 40.28 | 45.77 | 104.50 |  |
| 41 | 5.1.3.6 | Ribose 5-phosphate isomerase A | allA0556 | *RKI1* | 753 | 40.41 | 38.96 | 662.12 |  |
| 42 | 1.1.1.44 | 6-phosphogluconate dehydrogenase | allA4321 | *GND1* | 1479 | 583.61 | 542.16 | 1020.45 |  |
| 43 | 2.2.1.2 | Transaldolase | allA3126 | *TAL1* | 1005 | 495.57 | 683.86 | 816.08 |  |
| 44 | 2.7.1.15 | Ribokinase | allA4669 |  | 1008 | 4.45 | 3.23 | 29.12 |  |
| 45 | 1.11.1.6 | Catalase | allA1698 | *CTT1* | 1650 | 75.57 | 1449.22 | 303.31 | oxidative stress response |
| 46 | 1.8.1.9 | Thioredoxin reductase | allA4097 | *TrxR* | 960 | 2000.89 | 4020.98 | 372.98 |  |
| 47 | 1.8.1.7 | Glutathione reductase | allA4396 | *GLR* | 1410 | 28.65 | 150.03 | 52.76 |  |
| 48 | 1.11.1.9 | Glutathione peroxidase | allA3584 | *GPX* | 486 | 178.07 | 1077.13 | 211.99 |  |
| 49 | 1.15.1.1 | Superoxide dismutase | allA0410 | *SOD* | 465 | 585.14 | 848.19 | 479.07 |  |
| 50 |  | Thioredoxin | allA1233 | *TRX2* | 315 | 387.16 | 271.11 | 277.71 |  |
|  |  |  | allA3417 | *TRX3* | 450 | 151.85 | 84.75 | 100.43 |  |
| 51 | 1.11.1.15 | Peroxiredoxin (alkyl hydroperoxide reductase) | allA2475 |  | 594 | 7883.70 | 19909.68 | 2074.79 |  |
|  |  |  | allA2050 |  | 510 | 1771.20 | 809.78 | 359.87 |  |
| 52 |  | Major Facilitator Superfamily transporter | allA1940 | *RAG1/KHT1* | 1713 | 243.57 | 149.56 | 629.7 | transporters |
| 53 |  |  | allA1941 |  | 1689 | 337.99 | 27.69 | 112.28 |  |
| 54 |  |  | allA1937 | *KHT2* | 1701 | 111.28 | 65.63 | 138.4 |  |
| 55 |  |  | allA1938 |  | 1713 | 238.77 | 126.54 | 317.6 |  |
| 56 |  |  | allA1939 |  | 1698 | 127.34 | 282.86 | 350.28 |  |
| 57 |  |  | allA4236 | *HGT1* | 1689 | 111.48 | 62.53 | 762.20 |  |
| 58 |  |  | allA0822 | *FRT1* | 1716 | 194.02 | 103.62 | 745.55 |  |
| 58 |  |  | allA1964 |  | 1731 | 1605.30 | 9563.27 | 706.01 |  |
| 60 |  |  | allA3228 | *HXT8* | 1779 | 719.31 | 597.22 | 1093.19 |  |
| 61 |  |  | allA1121 | *STL1* | 1842 | 851.62 | 100.42 | 574.00 |  |
| 62 |  |  | allA3797 | *MIG1* | 1641 | 1308.69 | 223.36 | 496.34 | transcription regulation |
| 63 |  |  | allA2049 | *POP2* | 1362 | 19.59 | 24.65 | 56.33 |  |
| 64 |  |  | allA2704 | *CCR4* | 2451 | 18.82 | 12.04 | 95.10 |  |
| 65 |  | Heat shock protein | allA2000 | *HSP26* | 582 | 2710.83 | 6021.51 | 6492.27 |  |
| 66 |  |  | allA1820 | *HSP42* | 1185 | 1035.26 | 574.34 | 547.29 |  |
| 67 |  |  | allA0486 | *HSP31* | 714 | 453.04 | 2975.84 | 242.80 |  |
| 68 | 1.1.2.4 | D-lactate dehydrogenase | allA4131 |  | 1722 | 15.35 | 130.14 | 26.67 | others |
|  |  |  | allA1572 |  | 1797 | 28.31 | 89.06 | 365.19 |  |
|  |  |  | allA2485 |  | 1701 | 71.84 | 42.83 | 32.55 |  |
| 69 | 1.1.1.21 | D-xylose reductase | allA4095 | *XR* | 990 | 228.24 | 499.96 | 2101.06 |  |
| 70 | 2.4.1.15 | Trehalose 6-phosphate synthase | allA0655 | *TPS1* | 1518 | 111.06 | 58.08 | 204.67 |  |
| 71 | 3.1.3.12 | Trehalose 6-phosphate phosphatase | allA3387 | *TPS2* | 2652 | 55.20 | 125.74 | 161.92 |  |
| 72 | 3.2.1.28 | Neutral trehalase | allA1191 | *NTH1* | 2262 | 221.72 | 165.14 | 357.06 |  |
| 73 |  | Glycerol uptake facilitator protein | allA2816 |  | 930 | 426.12 | 60.11 | 526.73 |  |
| 74 | 2.7.1.30 | Glycerol kinase | allA0750 |  | 2007 | 65.98 | 74.71 | 615.22 |  |
| 75 | 2.7.1.47 | D-ribulokinase | allA2006 |  | 2340 | 230.96 | 2653.97 | 1829.55 |  |

**Table S3 Primes used in qPCR ana**lysis

| Gene Name | | Sequence | Length  (mer) | Gene Length  (bp) | Position  (mRNA) | Tm  （℃） | GC% | Product Length  (bp) |
| --- | --- | --- | --- | --- | --- | --- | --- | --- |
| KmINU1 | -F- | 5’- TACAACCCAGCAGCCACGAT -3’ | 20 | 1671 | 214 | 61.20 | 55.00 | 200 |
|  | -R- | 5’- GCTCTTTGTCTTGGGTCCAC-3’ | 20 |  | 413 | 56.8 | 55.00 |  |
| KmHXK1 | -F- | 5’-AGATTGCCAGACCACTTGAGAAC -3’ | 23 | 1458 | 337 | 61.92 | 47.83 | 144 |
|  | -R- | 5’-GGCTGGGTAAGAGAAGGTGAAA -3’ | 22 |  | 480 | 61.81 | 50.00 |  |
| KmGPM1 | -F- | 5’-GCTATCCAAACCGCCAACA -3’ | 19 | 744 | 181 | 61.96 | 52.63 | 93 |
|  | -R- | 5’- ACCGTAGTGTCTTTCGTTCAATCTC-3’ | 25 |  | 273 | 62.03 | 44.00 |  |
| KmFBP1 | -F- | 5’- TGTACGGTGCTTCCACTCACTT-3’ | 22 | 1056 | 539 | 62.03 | 50.00 | 218 |
|  | -R- | 5’-GCCATTTTCCTCACTCTTTTCCT -3’ | 23 |  | 756 | 62.07 | 43.48 |  |
| KmCTT1 | -F- | 5’-CAGCTAGAGACCCTAGAGGAGTGAG -3’ | 25 | 1650 | 320 | 61.37 | 56.00 | 131 |
|  | -R- | 5’-TTGCGTGTGGATAAAGTGTGG -3’ | 21 |  | 450 | 61.89 | 47.62 |  |
| KmGPD1 | -F- | 5’- GCTCCAGAAGTTGCTAAGGAAAAC-3’ | 24 | 1167 | 529 | 61.85 | 45.83 | 133 |
|  | -R- | 5’-CGTGGAAGTATGGTCTGTGGAA -3’ | 22 |  | 661 | 62.22 | 50.00 |  |
| KmACT1 | -F- | 5’-ACGTTGTTCCAATCTACGCC-3’ | 20 | 1137 | 491 | 57.60 | 50.00 | 195 |
|  | -R- | 5’-CTTGTTCGAAGTCCAAAGCG-3’ | 20 |  | 685 | 58.50 | 50.00 |  |
